# Applied Research on the Impact of a Neuromotor Development Program on the Lower Limb Strength of Junior Athletes in Greco-Roman Wrestling

**DOI:** 10.3390/sports13120428

**Published:** 2025-12-03

**Authors:** Florentin Vasilescu, Nicoleta Leonte, Cristiana Maria Porfireanu, Virgil Tudor

**Affiliations:** 1 Sportive Club No. 5, 010241 Bucharest, Romania; iuliu81@yahoo.co.uk; 2 Department of Physical Education and Sport—Physical Therapy, National University of Science and Technology Politehnica Bucharest, 060042 Bucharest, Romania; 3 Department of Physical Education and Sport, Academy of Economic Studies Bucharest, 010374 Bucharest, Romania; criss.porfi@gmail.com; 4 Faculty of Physical Education and Sport, National University of Physical Education and Sport, 060057 Bucharest, Romania; virgiltudor@yahoo.com

**Keywords:** explosive strength, juniors, Greco-Roman wrestling, intervention program, OptoJump

## Abstract

This study investigates the effects of a structured motor intervention program on the development of lower limb strength in junior athletes practicing Greco-Roman wrestling. Recognizing the crucial role of explosive strength in performing technical and decisive actions during combat, the research introduces a progressive, applied training protocol tailored to the neuromotor development of children aged 10 to 12 years (control group: M = 11.14, SD = 1.10; experimental group: M = 11.07, SD = 0.83). Conducted over 17 months, the study involved two groups of 14 registered wrestlers each from School Sports Club No. 5 in Bucharest. The experimental group participated in a complementary motor training program emphasizing plyometric drills, bodyweight strength exercises, and wrestling-specific movements, while the control group continued with the standard training routine. The intervention’s impact was evaluated using the OptoJump Next system, a biomechanical analysis tool measuring key indicators of explosive strength—jump height, ground contact time, flight time, and reactive strength index (RSI)—through the single-leg counter-movement jump (CMJ) test. Comparative analysis of pre- and post-intervention results showed significant improvements in neuromotor performance among athletes in the experimental group, confirming the effectiveness of the proposed methodology. This research thus provides a reproducible, evidence-based intervention model with direct applicability in optimizing the training of young Greco-Roman wrestlers.

## 1. Introduction

In Olympic wrestling—particularly in the Greco-Roman style—motor qualities such as explosive strength, reaction speed, and postural stability are fundamental to both technical and tactical performance. These attributes rely heavily on lower-body efficiency, which underpins balance, attack initiation, counteractions, and rapid phase transitions during combat [1,2].

The period of middle childhood and preadolescence (ages 10–12) represents an optimal window for neuromotor development interventions due to the heightened plasticity of the central nervous system and the increased adaptability of the musculoskeletal system to training stimuli [3,4]. At this stage, the foundation for reactive and explosive strength can be effectively developed through functional, age-appropriate methods that avoid excessive loading.

Recent studies have shown that well-structured and supervised plyometric training programs can significantly enhance young athletes’ neuromotor performance, directly influencing sport-specific abilities [5,6]. In Greco-Roman wrestling, lower-limb explosive strength is essential for executing rapid lifts, disengagements, blocks, and pivots from unstable positions. Therefore, developing this capacity during formative years has a lasting impact on athletic performance and helps prevent neuromuscular imbalances or injuries [7].

The assessment of these motor qualities has evolved from subjective evaluation toward the use of advanced technologies that provide precise, reproducible data. Among the most widely used tools in performance laboratories is the OptoJump Next system, which enables objective analysis of reaction speed, coordination, motor control, and neuromuscular balance. Based on optical sensors, the system allows biomechanical assessment of vertical jumps and other reactive movements. Measured parameters such as jump height, flight time, ground contact time, and the reactive strength index (RSI) serve as key indicators for evaluating lower-limb explosive power [2,8].

Contemporary literature further emphasizes the importance of tailoring motor interventions for junior athletes according to their individual profiles, developmental levels, and wrestling style. Experimental studies support the integration of plyometrics, bodyweight strength exercises, and sport-specific drills within a safe, progressive, and well-structured framework [1,9].

In wrestling, technical efficiency depends on the integration of multiple physical qualities. Explosive strength enables rapid attack initiation (projections, throws, leg entries), facilitates breaking grips and counterattacks, supports sudden changes in direction, and provides the energy “burst” needed at decisive moments. Lower-limb strength, as a combination of force and speed, generates the main impulse for lifts, projections, and pushes; ensures stability in defensive and ground positions; and maintains pressure in clinches or edge-of-mat situations. Balance allows athletes to resist pushes and pulls without losing position, recover quickly after directional changes, and maintain control during throws. Coordination ensures smooth transitions between techniques (e.g., attack to counterattack), accuracy in pivots and releases, and efficient movement that minimizes unnecessary energy expenditure.

This study aligns with current scientific approaches by proposing an applied neuromotor development program aimed at enhancing lower-limb explosive strength in junior Greco-Roman wrestlers [10,11]. The 17-month intervention was objectively evaluated through repeated OptoJump testing, providing a solid quantitative basis for assessing the effectiveness of the applied methodology.

The research seeks to strengthen the scientific foundation of training for junior athletes by offering coaches a validated, reproducible model with practical applicability. It also presents an integrated perspective on how explosive strength can be safely and effectively developed and monitored without compromising the health or natural growth of young wrestlers.

The study is guided by two central research questions:

What is the effect of a specific intervention program on the development of lower-limb explosive strength in junior Greco-Roman wrestlers, as measured by the OptoJump system?

Which training methods (exercises and techniques) are most effective for optimizing lower-limb explosive strength in young wrestlers, considering their age and the demands of the Greco-Roman style?

To address these questions, the study is based on the hypothesis that implementing a structured neuromotor development program—centered on plyometric exercises, functional strength training, and wrestling-specific applications—will produce significant improvements in the lower-limb explosive strength of junior athletes compared to those following a standard training regimen. The effectiveness of this program is expected to be demonstrated through objective data obtained from the OptoJump testing system, revealing statistically significant gains in the experimental group relative to the control group.

## 2. Materials and Methods

### 2.1. Participants

#### Research Conditions

The participants included in this experimental research were organized into two distinct groups of junior athletes: an experimental group (EG) and a control group (CG), each composed of 14 subjects (*n* = 14). These athletes, aged between 10 and 12 years, were registered and actively training in the Greco-Roman wrestling section of School Sports Club No. 5 in Bucharest (Table 1).

The choice of this size took into account:

A. Available population and selection criteria

The study took place within the School Sports Club no. 5 Bucharest, the Greco-Roman wrestling section, where the number of athletes aged 10–12 years and comparable level of training was limited. The inclusion of 28 subjects allowed coverage of the entire eligible population, while maintaining the practical relevance of the research.

B. Randomisation to remove bias

After identifying the eligible athletes, they were randomly assigned (randomised) to the two groups using a draw procedure.

Randomization was designed to reduce the influence of subjective factors (e.g., coach preferences, past performance) and increase the internal validity of the study.

In this way, the initial differences between the groups have been minimised and any observed effects can be attributed with greater confidence to the experimental programme.

To be eligible, athletes had to meet all of the following conditions:

- Age between 10 and 12 years at the start of the study.
- Active membership in the Greco-Roman wrestling section of School Sports Club No. 5 Bucharest, with regular attendance at training sessions.
- Minimum training experience (e.g., at least 6 months–1 year) to ensure a basic level of technical and motor skills.
- Good health status, confirmed by a sports medical certificate, with no conditions that would prevent participation in high-intensity physical effort.
- Informed consent from parents/legal guardians for participation in the study.

Exclusion Criteria Athletes were excluded if they met any of the following conditions:

- Acute or chronic musculoskeletal injuries, or other medical conditions limiting physical activity (e.g., cardiac, respiratory problems).
- Frequent absences from training sessions, which could affect the implementation of the experimental program.
- Concurrent participation in other special training programs or competitions that might influence the studied variables.
- Lack of informed consent from parents/legal guardians.
- Failure to meet age criteria or club membership requirements.

The two groups were carefully balanced in terms of initial morphological, functional, and motor characteristics to ensure optimal homogeneity. This homogeneity was essential for objectively conducting the experimental intervention and created favorable conditions for comparing the effects of the specific intervention program applied to the experimental group with those of the standard training program followed by the control group. Additionally, age, height, weight, and body mass index (BMI) indicators of the children in both research groups were analyzed. All subjects were declared medically fit for specific physical effort, according to their sports medical records. Their participation in the study was conducted in strict compliance with current ethical standards regarding research involving minors. The experimental protocol was approved by the Ethics Committee of School Sports Club No. 5 Bucharest (499/11 June 2025), and informed consent was obtained in writing from the athletes’ parents or legal guardians before the commencement of any research procedures.

### 2.2. Study Design

The research was carried out at School Sports Club No. 5 in Bucharest, within a controlled environment specifically adapted to the training requirements of junior Greco-Roman wrestlers. The experimental procedures were conducted under real training conditions, while maintaining the methodological rigor characteristic of a systematically planned intervention study.

The independent variable—the specific intervention program—was applied to the experimental group over a period of approximately 17 months, from March 2023 to September 2024 (see Table 2). During this time, athletes in the experimental group participated in a structured training program designed to complement the classical training method, with a particular focus on developing lower-limb explosive strength. In contrast, the control group continued to follow the club’s standard training regimen, without any additional exercises or interventions related to the research objectives.

Throughout the study, all working conditions were carefully controlled. The sports infrastructure (wrestling mats, strength training equipment), training schedule, and coaching staff remained constant for both groups to minimize the potential influence of external variables. Both the initial and final assessments were conducted under identical conditions—within the same training facility and using the same technological equipment (OptoJump Next system)—to ensure the validity, reliability, and comparability of the collected data.

### 2.3. Research Content

The present research followed an applied experimental design aimed at evaluating the influence of a specific neuromotor development program on lower-limb strength in two groups of junior athletes practicing Greco-Roman wrestling. The intervention program was structured to enhance explosive strength and motor control through the integration of plyometric exercises and wrestling-specific techniques.

The research process was organized into four main stages:

1. Documentation and Research Design Stage

During this initial stage, recent specialized literature was analyzed to establish the theoretical foundation of the study. The research hypothesis and objectives were formulated, the intervention methods were defined, and the biomechanical indicators relevant for evaluating lower-limb strength were selected.

2. Initial Evaluation Stage (Pretesting)

The pretesting phase took place at the beginning of the experimental period (March 2023) at the training facility of School Sports Club No. 5 in Bucharest. All participating athletes were evaluated using the OptoJump Next system, performing the countermovement jump (CMJ) to establish baseline measurements for jump height, ground contact time, and reactive strength index (RSI).

Previous studies have reported a coefficient of variation (CV%) from 1 to 5% and an intra-class correlation coefficient (ICC) greater than 0.90 for test–retest assessments using this system, confirming its high accuracy and reliability in evaluating neuromotor performance.

3. Applied Intervention Stage

The intervention phase was conducted over approximately 17 months (March 2023–September 2024) and involved the implementation of a progressive neuromotor development program applied exclusively to the experimental group.

The decision to extend the program to 17 months was based on two primary considerations:

Gradual and safe adaptation: Adequate time was needed for the athletes to progressively develop fundamental motor capacities—strength, speed, endurance, and mobility—while minimizing overload and injury risk.

Structured periodization: The 17-month duration allowed for division into macrocycles (annual) and mesocycles (monthly or bimonthly), ensuring a logical progression through the main phases of preparation: general, specific, competitive, and transition training.

The program incorporated plyometric exercises, wrestling-specific movements aimed at lower-limb strength development, and functional activities aligned with the technical demands of the Greco-Roman style. The control group, in parallel, continued their standard club training program without additional interventions related to the research objectives.

All training sessions were conducted at the facilities and laboratories of School Sports Club No. 5 in Bucharest under continuous supervision and methodological control.

4. Final Evaluation Stage (Post-testing)

The post-testing phase was carried out at the end of the intervention period (September 2024) under identical conditions to the pretest. The same equipment, testing protocols, and evaluation procedures were applied to ensure consistency and data comparability.

The collected results were statistically analyzed to determine the effectiveness of the intervention and to identify performance differences between the experimental and control groups.

Through this structured approach, the research seeks to provide scientific validation for a specific intervention program aimed at developing lower-limb strength in junior Greco-Roman wrestlers. By utilizing objective, technology-assisted evaluation methods, the study contributes to strengthening the methodological foundation of physical training for this age category and offers practical insights for optimizing athletic development in Greco-Roman wrestling.

#### 2.3.1. Training Methods Used in the Research

Experimental Group—Intervention Focused on Lower Limb Strength Development and Standing Wrestling Techniques

The program designed for the experimental group targeted specific neuromotor development, directly enhancing lower limb explosiveness, reactivity, and stability through a functional integration of strength components into wrestling techniques performed in a standing position. Accordingly, the program included advanced plyometric exercises, functional partner training, tactical and technical applications, and specialized thematic sequences for Greco-Roman wrestling.

Specific Plyometric Exercises were aimed at increasing reactive capacity and explosive strength, including:

- Jumps on a mat from a guard position
- Drop jumps with immediate takeoff and repositioning for engagement
- Lateral jumps over a partner, followed by rapid penetration into attack position
- Squat jumps combined with partner lifting exercises

##### Functional Strength Training Included

- Squats with a partner on the back, for posterior chain development and stability
- Pulls and pushes from a clinch position
- Repeated mannequin lifts using a belt grip
- Circuit exercises incorporating “sumo walks,” lunges with grip, and balance drills simulating wrestling movements

##### Technical-Tactical Elements Specific to Greco-Roman Wrestling

- Takedowns via belt grip, lifting, and swinging, emphasizing force impulse synchronization
- Rotations from torso grasp, placing opponents in disadvantageous positions
- Low-guard penetrations, adjusting center of gravity and efficient lower limb positioning
- Thematic applications where athletes executed techniques immediately after securing a grip

##### Reactive and Stability Components Were Developed Through

- Tandem exercises (pushes, resistance, holding)
- Repetitions of low-guard throws using mannequins
- Medicine ball drills (e.g., vertical and lateral throws from strength positions)
- Video feedback and biomechanical corrections regarding lower limb positioning and technique execution

This set of methods aimed to optimize physical qualities and wrestling-specific motor skills simultaneously, ensuring a real transfer between neuromotor development and competitive performance in Greco-Roman wrestling (see Table 3).

##### Control Group—Standard Training Program for the Greco-Roman Style

The control group was trained using a traditional program commonly applied in beginner or intermediate-level athlete preparation, without biomechanical analysis or technology-assisted interventions. The training focused on learning and reinforcing fundamental techniques of the Greco-Roman style using conventional general and specific training methods.

##### General Physical Component Included

- Linear runs and obstacle runs, push-ups, sit-ups, pull-ups
- Basic squats, stationary or dynamic jumps, mobility drills

Technical Exercises Focused On:

- Learning and repeating takedown techniques such as basic belt grip and balanced throws
- Controlled projections with a partner, without explosive or reactive elements
- Mannequin drills to reinforce fundamental techniques in takedowns and lifts

Tactical Applications Were Conducted Through

- Thematic training fights (engagements without ground transition, responses to slow attacks)
- Attack/counterattack simulations from fixed guard positions or in restricted spaces
- Paired drills in repeated series, without progressive difficulty increments

Motor Skill Games Targeted:

- Balance maintenance, controlled opposition, gripping and postural stability exercises
- Wrestling-themed games adapted to the athletes’ age

This program ensured a standard training framework without additional optimization elements, making it ideal for comparing the effectiveness of the intervention applied to the experimental group.

#### 2.3.2. Research Variables

In the experimental research conducted at School Sports Club No. 5 in Bucharest, two groups of young junior athletes followed differentiated training programs designed to examine the impact of neuromotor development on lower-limb strength parameters. The methodological design involved the application of an independent variable to the experimental group, while the control group continued with the standard training program, in order to highlight potential functional and motor adaptations.

The independent variable was represented by the neuromotor intervention program applied to the experimental group. This program integrated plyometric exercises, functional strength development methods, and standing wrestling techniques specific to the Greco-Roman style. It was designed to stimulate the development of explosive strength and lower-limb reactive capacity, and was carefully adapted to the athletes’ age and training level.

The dependent variable consisted of the level of lower-limb strength development, objectively measured using the OptoJump Next system through countermovement jump (CMJ) testing. The analyzed indicators included jump height, flight time, ground contact time, and the Reactive Strength Index (RSI), which reflects neuromuscular efficiency and the ability to generate force rapidly.

By analyzing these variables, the research aims to provide an objective foundation for evaluating the effectiveness of a targeted strength development program within a specific wrestling context. Ultimately, the study seeks to contribute to the scientific substantiation of methodological decisions in the training of young performance athletes.

#### 2.3.3. Statistical Analysis

The data were analyzed using the Statistical Package for the Social Sciences (SPSS, version 29.0.2.0; IBM, Armonk, NY, USA). The data collected from various measurements and tests were subjected to statistical processing using the following procedures and indicators.

For group comparisons, Analysis of Variance (ANOVA) and dependent-samples *t*-tests were applied where appropriate. For ANOVA, F-values, *p*-values, and effect sizes (η^2^) were reported, while for dependent-samples *t*-tests, t-values and *p*-values were presented. In addition, Pearson correlation coefficients (r) were calculated to examine relationships between key variables.

The normality of data distributions was verified using the Shapiro–Wilk test, and the homogeneity of variances was assessed. All assumptions required for the applied statistical tests were satisfied.

The sample size was determined a priori using power analysis (G*Power, version 3.1.9.8). Assuming a medium effect size (f = 0.25), an alpha level of 0.05, and a statistical power of 0.80, the required sample size was calculated as N = 28 participants. Accordingly, the study included 28 junior athletes (14 in the experimental group and 14 in the control group), meeting the minimum sample size requirement [12,13].

## 3. Results

**Figure 1:**
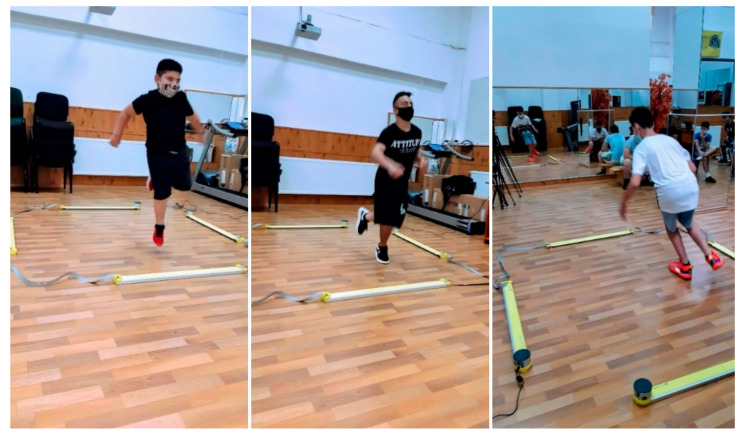
Images of OptoJump Next Testing.

During testing with the OptoJump analysis system, vertical jumps (5JS2D) were performed, with five repetitions for each leg (right and left). These measurements provided conclusive data for our research (Figure 1).

Five vertical jumps were performed for each lower limb, with the OptoJump computerized program objectively measuring the following indicators:

- T Cont—ground contact time with the force plate, measured in fractions of a second
- T Flight—flight time (time spent in the air), measured in fractions of a second
- Height—jump height (calculated in cm)
- RSI—Reactive Strength Index—“The reactive strength index (RSI) is an effective marker of reactive force due to the rapid shortening caused by prior activation in the DJ—Drop Jump, also known as the stretch-shortening cycle” [10].
- Pace—jump cadence
- Power—strength (watts/kg)

We considered vertical jump performance to be the most relevant indicator for Greco-Roman wrestling, given the specificity of techniques in this discipline. For this reason, we conducted all comparative analyses between the subjects of the two research groups based on this parameter.

### 3.1. Descriptive Statistics

**Control Group vs. Experimental Group**

The program produced major improvements in height and power variables (the core performance parameters), with a large effect size and a clear Group × Time interaction; the Experimental group progressed significantly more than the Control group.

For Tcont, TFlight, and RSI variables, moderate effects were observed (the Experimental group shows a better tendency, but not as strong).

Pace variable remained stable → it was not affected by the intervention (see Table 4).

#### Inferential Statistics

*T*-Test—Control Group versus Experimental Group (Final Testing)

In order to find out whether there is a statistically significant difference between the initial and the final *t*-test, we apply the Paired Two Sample for Means (*t*-Test).

- Ground Contact Time (GCT):

The t-statistic obtained for this test was t(26) = 2.71, exceeding the two-tailed critical t-value (1.67) at a 95% confidence level. The result was statistically significant (*p* = 0.01 < 0.05). The mean value of GCT in the control group (0.55 s) was higher than that of the experimental group (0.37 s), indicating that the experimental training program effectively reduced ground contact duration. The differences between individual results and group means were statistically significant, confirming the robustness of this finding.

- Flight Time (FT):

For Flight Time, the test produced t(26) = 3.69, also exceeding the critical t-value (1.67) with a high level of significance (*p* < 0.001, 95% confidence). The mean value in the control group (0.21 s) was lower than that in the experimental group (0.29 s). This statistically significant difference supports the research hypothesis, indicating improved explosive performance among athletes in the experimental group.

- Group Consistency:

The control group displayed a higher standard deviation (SD = 0.12), reflecting greater variability in performance, whereas the experimental group showed a lower SD (0.01) and a very small coefficient of variation (CV = 0.01), demonstrating high internal consistency and stability of results.

Overall, the inferential statistical tests confirm that t_stat_ > t_(Crit)_ and *p* < 0.05 for the analyzed parameters, allowing the results to be generalized to the broader population of junior Greco-Roman wrestlers. In other words, if the tests were repeated under similar conditions, comparable outcomes would be expected (see Table 5).

Based on the values of standard deviation (SD = 8.30; 11.56) and coefficient of variation (CV = 68.95; 133.7), the results of the control group showed substantial dispersion, indicating considerable variability in individual performances for both jump height and power. In contrast, the experimental group demonstrated higher and more consistent performance levels, reflecting improved homogeneity and training adaptation.

- Jump Height:

The test yielded t(26) = 2.10, which exceeds the two-tailed critical t-value (1.67) at the 95% confidence level. The result was statistically significant (*p* = 0.01 < 0.05). The mean jump height of the control group (7.61 cm) was lower than that of the experimental group (11.60 cm). This significant difference confirms the research hypothesis, indicating that the neuromotor intervention program effectively enhanced vertical jump performance.

- Power per kg of Body Weight:

For this indicator, the test produced t(26) = 1.21, which is below the two-tailed critical t-value (1.67). The result was not statistically significant (*p* = 0.15 > 0.05) at the 95% confidence level. Although the experimental group showed a higher mean power output (13.50 W/kg) compared to the control group (10.52 W/kg), the difference cannot be generalized to the entire population since t_stat_ < t_(Crit)_ and *p* > 0.05.

Nevertheless, even statistically non-significant differences in explosive power can have practical importance in Greco-Roman wrestling, where subtle variations in force application often influence the outcome of competitive matches. These findings are summarized in Table 6.

- Jump Frequency (Cadence):

The test produced t(26) = 0.10, which is below the two-tailed critical t-value (1.67) at the 95% confidence level. The result was not statistically significant (*p* = 0.15 > 0.05). The mean jump frequency for the control group (1.56 jumps/s) was nearly identical to that of the experimental group (1.57 jumps/s), indicating no meaningful difference between the groups.

- Reactive Strength Index (RSI):

For the RSI, the test yielded t(26) = 0.56, which is also below the two-tailed critical t-value (1.68), with *p* = 0.15 > 0.05. The mean RSI in the control group (0.27) was slightly lower than that in the experimental group (0.32); however, this difference was not statistically significant.

Since t_stat_ < t_(Crit)_ and *p* > 0.05 for both indicators, the inferential statistical tests do not allow these results to be generalized to the broader population. In other words, if the tests were repeated under similar conditions, comparable results might not be consistently observed.

- Two-Sample *t*-Test Summary:

When comparing the control and experimental groups, the two-sample *t*-test assuming equal variances revealed statistically significant differences for Ground Contact Time, Flight Time, and Jump Height. In contrast, no significant differences were found for Power, Jump Frequency (Cadence), or the Reactive Strength Index.

These findings indicate that the neuromotor intervention program applied to the experimental group had a stronger and statistically validated effect on explosive movement parameters (contact time, flight time, and jump height). Although notable numerical differences were observed for power, cadence, and RSI, they did not reach statistical significance. This suggests that while the training program improved several key aspects of explosive performance, further research is warranted to optimize its effects on power output and reactive strength (Table 7).

### 3.2. Statistical Association

**Correlation Coefficient—Control Group**

A strong positive correlation was observed among the following indicators: Flight Time, Jump Height, Power per kg of body weight, and Reactive Strength Index (RSI) (Table 8). All these parameters represent distinct expressions of lower-limb force production.

- Flight Time and Jump Height:

A very strong positive correlation was found between Flight Time and Jump Height (r = 0.90). In addition, Flight Time showed strong correlations with Power per kg of body weight (r = 0.81) and with the Reactive Strength Index (r = 0.72). Practically, this indicates that athletes who exhibit longer flight times also tend to achieve greater jump heights and higher levels of power output and reactive strength.

- Jump Height and Power/Reactive Strength Index:

Jump Height demonstrated a very strong positive correlation with both Power per kg of body weight (r = 0.92) and the Reactive Strength Index (r = 0.88). These findings suggest that athletes capable of achieving greater jump heights also display higher power output and greater reactive strength, emphasizing the interdependence of these performance indicators.

- Power per kg Body Weight and Reactive Strength Index:

An almost perfect positive correlation was identified between Power per kg of body weight and RSI (r = 0.99), indicating that increases in relative power are almost directly proportional to increases in reactive strength. This reinforces the notion that both variables capture closely related aspects of explosive neuromuscular performance.

- Jump Frequency and Ground Contact Time:

In contrast, a moderate negative correlation was found between Jump Frequency and Ground Contact Time (r = −0.67), suggesting that higher jump frequencies are associated with shorter ground contact times—a desirable feature in explosive athletic movements.

- Jump Frequency and Flight Time/Jump Height:

A small negative correlation was observed between Jump Frequency and both Flight Time (r = −0.42) and Jump Height (r = −0.42). This relationship is expected, as increases in Flight Time and Jump Height (indicating greater power generation) are typically accompanied by a reduction in jump cadence. Conversely, higher jump frequencies are associated with shorter flight times and lower jump heights, reflecting a trade-off between frequency and power expression (Table 8).

**Correlation Coefficient—Experimental Group**

In Table 9 a strong positive correlation is observed between:

- Flight Time and Height (r = 0.98), Flight Time and Power (r = 0.98), and Flight Time and Reactive Strength Index (r = 0.96): This indicates that high values of Flight Time are associated with high values of Height, Power, and the Reactive Strength Index.
- Height and Power (r = 0.98) and Height and Reactive Strength Index (r = 0.97): In other words, high jump heights are associated with high power output and high values of the reactive strength index (with a correlation of r = 0.99 reported in one instance).
- Power and Reactive Strength Index (r = 0.99): Very high power values are, therefore, almost perfectly associated with very high Reactive Strength Index values.

A moderate positive correlation is observed between:

- Jump Cadence and Reactive Strength Index (r = 0.50): This means that higher jump cadence values tend to be associated with moderate increases in the Reactive Strength Index.

On the other hand, there is a small negative correlation—without statistical significance—between:

- Ground Contact Time and Flight Time (r = −0.32)
- Ground Contact Time and Height (r = −0.30)
- Ground Contact Time and Power (r = −0.37)
- Ground Contact Time and Jump Cadence (r = −0.37)
- Ground Contact Time and Reactive Strength Index (r = −0.40)

These negative correlations indicate that longer Ground Contact Times are associated with lower values of Flight Time, Height, Power, Jump Cadence, and the Reactive Strength Index. In practical terms, when the expression of force (in the form of power) is lower, Ground Contact Time tends to be higher.

## 4. Discussion

This study contributes to the specialized literature on optimizing the physical and technical-tactical training of junior Greco-Roman wrestlers by introducing an intervention program focused on developing lower-body strength and executing techniques in a standing position. A distinctive feature of this research is the use of the OptoJump system for precise quantification of neuromotor performance, providing an objective evaluation framework [14,15,16].

According to recent studies, the performance of Greco-Roman wrestlers is closely linked to explosive strength and reaction ability, particularly in executing offensive maneuvers initiated from a standing position. These components are essential in an acyclic sport characterized by rapid alternation between maximum-intensity efforts and phases of control or energy conservation [17,18,19,20]. The findings of our research, which showed a significant increase in jump height and the Reactive Strength Index (RSI) in the experimental group, suggest that these characteristics may play a relevant role in athletic performance, although their direct contribution to competitive success requires further investigation.

The specific effort required in Greco-Roman wrestling places continuous demands on the neuromuscular system under conditions of constant contact with the opponent, where reaction speed and motor coordination are decisive factors [21,22]. Therefore, the selection of training methods such as plyometric jumps, dummy lifts, and clinch-position exercises facilitated a direct transfer between physical development and the application of sport-specific techniques.

Mocanu et al. (2023) [23] emphasize that junior competitive performance is determined not only by general physical preparation but also by its integration into tactical contexts similar to competition scenarios.

Furthermore, the homogeneity of performance within this group, observed in statistical analysis, aligns observations on the positive effects of sport-specific adapted programs in standardizing performance [24].

An additional explanation of the observed progress lies in the differentiated methodological approach. While the control group followed a conventional training regimen focused on general exercises and fundamental techniques, the experimental group benefited from functional stimuli tailored to competition demands. This methodological distinction also explains the more modest progress recorded by the control group, a finding supported by Nagovitsyn et al.’s (2019) [25] research on the necessity of adapting training programs to the neuromotor profile of young wrestlers.

Moreover, recent studies indicate that utilizing technological measurement systems such as OptoJump not only ensure objectivity but also enhance athletes’ motivation and engagement in the learning process [26,27,28,29]. Although no direct questionnaire-based evaluation was conducted, this observation is indirectly supported by the behavior of the athletes in the experimental group, who consistently demonstrated active engagement and a more rapid adaptation to the technical and physical demands of training. This indirect evidence should, however, be interpreted with caution, as subjective measures of perception and motivation were not systematically collected.

Plyometric exercises combined with wrestling techniques, along with the inclusion of tactical sequences under opposition conditions, have facilitated a genuine transfer between the development of neuromotor qualities and their applicability in actual combat. The effectiveness of this training approach is supported by recent literature, which advocates for aligning physical preparation with the biomechanical and tactical demands of combat sports [30,31,32,33]. Additionally, integrated training methods that incorporate complex motor tasks within decision-making contexts have been shown to contribute to the development of advanced neuromotor control and the execution efficiency of techniques under competitive conditions [34,35,36,37].

In this context, it is essential to highlight that neuromotor development is not merely a consequence of increased exercise volume but, more importantly, of the quality of these stimuli and how they are integrated into the technical-tactical content specific to Greco-Roman wrestling [38,39,40,41].

Limits. While the findings provide valuable insights into the effectiveness of a structured motor intervention program for junior Greco-Roman wrestlers, several limitations should be acknowledged. First, the sample size was relatively small (n = 28), which may limit the generalizability of the results to larger or more diverse populations. Second, the participants were recruited from a single sports club, meaning that contextual factors such as coaching style, training culture, and facilities may have influenced the outcomes. Third, although the intervention spanned 17 months, longer-term follow-up was not conducted, so it remains unclear whether the observed improvements are maintained over time. Finally, the study focused primarily on explosive strength parameters (e.g., CMJ performance, RSI, flight and contact times), while other relevant dimensions of athletic performance, such as technical execution under fatigue or injury risk, were not assessed.

## 5. Conclusions

The study conducted on the impact of a structured neuromotor development program has highlighted significant advancements in lower-limb power among junior Greco-Roman wrestlers. Over a 17-month period, the experimental group, which engaged in a specialized training regimen that included plyometric exercises, body-weight strength applications, and wrestling-specific drills, showed marked improvements in key explosive power metrics. These results underscore the effectiveness of the proposed intervention model, providing coaches and trainers with a reproducible approach to optimize the training of young wrestlers. Future research should continue to explore the long-term benefits of such targeted training programs across various age groups and competitive levels in wrestling. The originality of this research lies in its integrative approach, which combines neuromotor development with sport-specific applications in Greco-Roman wrestling for young junior athletes. Furthermore, the research employs objective biomechanical monitoring tools, such as the OptoJump system and video feedback, to quantify performance changes and refine technical execution. By contrasting this with a control group following conventional methods, the study highlights the efficacy of the intervention and provides a practical, replicable methodological framework for coaches to support the athletic development of young wrestlers. This represents both the scientific innovation and applied expertise of the research.

## Figures and Tables

**Table 1:** Subjects of the Research Groups (Control and Experimental)—Anthropometric Parameters in the Initial Phase of the Study.

Variables	Mean		Standard Deviation	
	**CG** *	**EG** *	**CG** *	**EG** *
Age (ani)	11.14	11.07	1.10	0.83
Body mass (kg)	53.79	54.64	13.71	18.85
Height (cm)	154.57	156.86	13.19	13.92
BMI	22.4	20.8	3.58	5.1
**Variable**	**t-statistic**	**df**	***p*-value**	
Age	0.19	24.18	0.851	
Body mass	−0.14	23.75	0.893	
Height	−0.45	25.92	0.659	
BMI	0.96	23.31	0.347	

CG *—Cotrol group. EG *—Experimetal group. All *p*-values > 0.05, meaning no statistically significant differences exist between the control and experimental groups at baseline for age, body mass, height, or BMI. The groups can be considered comparable before the intervention.

**Table 2:** Research Stages.

Period	Activity
March 2023	Initial Testing
April 2023–August 2024	Implementation of the Intervention Program
September 2024	Final Testing

**Table 3:** The weekly training program.

Day/Focus	Warm-Up (10–15 Min)	Plyometrics	Functional Strength	Technical–Tactical	Reactive & Stability	Session Duration
Day 1— Explosiveness	Dynamic mobility, wrestling drills	Jumps from guard 3 × 10 Drop jumps 3 × 8 Lateral jumps + penetration 3 × 6/side Squat jumps + partner lift 3 × 8	Squats w/partner 3 × 10 Mannequin lifts 3 × 6	Belt-grip takedowns 4 × 5 Torso rotations 3 × 6	–	~75–80 min
Day 2—Strength & Stability	Dynamic warm-up, balance drills	Jumps from guard 2 × 10	Squats w/partner 3 × 10 Clinch pulls/pushes 3 × 20 s Circuit: sumo walks, lunges w/grip, balance (3 rounds, 30 s each, 30 s rest)	Low-guard penetrations 3 × 8	Tandem pushes/pulls 3 × 20 s Low-guard mannequin throws 3 × 6 Med ball throws (vertical 3 × 8, lateral 3 × 8/side)	~80–90 min
Day 3—Integration & Simulation	Wrestling-specific mobility, dynamic partner drills	Drop jumps 3 × 8 Lateral jumps + penetration 3 × 6/side	Circuit: sumo walks, lunges, balance (3 rounds, 30 s/30 s)	Thematic sequences: grip → immediate technique 4 × 30 s Belt-grip takedowns 3 × 5	Tandem drills 3 × 20 s Med ball throws 3 × 8 each	~80–90 min

**Table 4:** Statistical indicators—Control Group and the Experimental Group in the Initial and Final Testing.

Variable	Group	IT *** (Mean ± SD)	FT *** (Mean ± SD)	ANOVA (Time, Group)	η^2^	Interpretation
TCont.	CG *	0.53 ± 0.19	0.63 ± 0.36	F(1,26) = 4.8, *p* = 0.038 (T) ns (G)	0.16	GC increases, EG decreases at FT
	EG *	0.49 ± 0.20	0.42 ± 0.15			
TFlight	CG	0.16 ± 0.07	0.22 ± 0.10	F(1,26) = 6.4, *p* = 0.018 (T) ns (G)	0.12	Both groups increase, more in EG
	EG	0.14 ± 0.05	0.28 ± 0.09			
Height	CG	4.2 ± 2.5	11.7 ± 8.0	F(1,26) = 45.2, *p* < 0.001 (T) F(1,26) = 5.8, *p* = 0.024 (G)	0.36	Large effect, clear progress in EG
	EG	2.8 ± 1.5	12.6 ± 5.4			
Power	CG	6.1 ± 5.3	14.2 ± 12.0	F(1,26) = 39.1, *p* < 0.001 (T) F(1,26) = 6.2, *p* = 0.020 (G)	0.34	Strong increase, EG improves more
	EG	4.9 ± 2.1	13.9 ± 5.0			
Pace	CG	1.65 ± 0.30	1.60 ± 0.30	ns ** (T, G)	<0.05	Stable variable, no changes
	EG	1.74 ± 0.28	1.55 ± 0.19			
RSI	CG	0.12 ± 0.08	0.31 ± 0.28	F(1,26) = 5.9, *p* = 0.022 (T) ns (G)	0.14	Small increase, more accentuated in EG
	EG	0.07 ± 0.03	0.34 ± 0.17			

CG, EG *—control group, experimental group. ns **—not significant. FT, IT ***—Final test, Initial Test.

**Table 5:** Statistical indicators –Ground Contact Time and Flight Time (CG vs. EG).

Indicators		Mean	SD	CV	t Stat	P (T ≤ t) One-Tail	t Critical One-Tail	P (T ≤ t) Two-Tail	t Critical Two-Tail
Ground Contact Time	CG * EG **	0.55	0.34	0.12	2.71	0.00	1.67	0.01	2.01
		0.37	0.09	0.01					
Flight Time	CG EG	0.21	0.09	0.01	−3.69	0.00	1.67	0.00	2.01
		0.29	0.07	0.01					

CG *—control group; EG **—experimental group.

**Table 6:** Statistical indicators—Height and Power (CG vs. EG).

Indicators		Mean	SD	CV	t Stat	P (T ≤ t) One-Tail	t Critical One-Tail	P (T ≤ t) Two-Tail	t Critical Two-Tail
Height	CG EG	7.61	8.30	68.95	−2.10	0.02	1.67	0.04	2.01
		11.60	1.50	2.24					
Power	CG EG	10.52	11.56	133.70	−1.21	0.11	1.67	0.23	2.01
		13.50	1.76	3.09					

**Table 7:** Statistical indicators –Cadence and Reactive Strength Index—RSI (CG vs. EG).

Indicators		Mean	SD	CV	t Stat	P (T ≤ t) One-Tail	t Critical One-Tail	P (T ≤ t) Two-Tail	t Critical Two-Tail
Cadence	CG EG	1.56	0.37	0.14	−0.10	0.46	1.67	0.92	2.01
		1.57	0.18	0.03					
Reactive Strength Index (RSI)	CG EG	0.27	0.50	0.24	−0.56	0.29	1.68	0.58	2.01
		0.32	0.16	0.03					

**Table 8:** Correlation between Variables—Control Group.

Control	Test	T Cont.	T Flight	Height	Power	Pace	RSI
Test	1.00						
T Cont.	0.04	1.00					
T Flight	0.37	−0.03	1.00				
Height	0.33	−0.08	0.90	1.00			
Power	0.29	−0.16	0.81	0.92	1.00		
Pace	−0.08	−0.67	−0.42	−0.31	−0.20	1.00	
RSI	0.26	−0.19	0.72	0.88	0.99	−0.13	1.00

**Table 9:** Correlation between Variables—Experimental Group.

Experiment	Test	T Cont.	T Flight	Height	Power	Pace	RSI
Test	1.00						
T Cont.	−0.37	1.00					
T Flight	0.81	−0.32	1.00				
Height	0.78	−0.30	0.98	1.00			
Power	0.78	−0.37	0.98	0.98	1.00		
Pace	−0.35	−0.37	−0.63	−0.58	−0.57	1.00	
RSI	0.76	−0.40	0.96	0.97	0.99	−0.50	1.00

## Data Availability

Supporting raw data for the conclusions drawn in this article are available from the authors on demand.

## Abbreviations

The following abbreviations are used in this manuscript:

CG	Control group
EG	Experimental group
** ns	not significant
*** FT, IT	Final test, Initial Test
